# Adaptive response to iterative passages of five *Lactobacillus* species in simulated vaginal fluid

**DOI:** 10.1186/s12866-020-02027-8

**Published:** 2020-11-10

**Authors:** Katelyn Brandt, Rodolphe Barrangou

**Affiliations:** 1 Functional Genomics Graduate Program, North Carolina State University, Raleigh, NC 27695 USA; 2 Department of Food, Bioprocessing and Nutrition Sciences, North Carolina State University, Raleigh, NC 27695 USA

**Keywords:** *Lactobacillus*, Vaginal, Passages, Probiotics

## Abstract

**Background:**

Microbiome and metagenomic studies have given rise to a new understanding of microbial colonization of various human tissues and their ability to impact our health. One human microbiome growing in notoriety, the vaginal microbiome, stands out given its importance for women’s health, and is peculiar in terms of its relative bacterial composition, including its simplicity and typical domination by a small number of *Lactobacillus* species. The loss of *Lactobacillus* dominance is associated with disorders such as bacterial vaginosis, and efforts are now underway to understand the ability of *Lactobacillus* species to colonize the vaginal tract and adapt to this dynamic and acidic environment. Here, we investigate how various *Lactobacillus* species often isolated from the vaginal and intestinal cavities genomically and transcriptionally respond to iterative growth in simulated vaginal fluid.

**Results:**

We determined the genomes and transcriptomes of *L. acidophilus, L. crispatus, L. fermentum, L. gasseri,* and *L. jensenii* and compared profiles after 50, 100, 500, and 1000 generations of iterative passages in synthetic vaginal fluid. In general, we identified relatively few genetic changes consisting of single nucleotide polymorphisms, with higher counts occurring more frequently in non-vaginal isolated species. Transcriptional profiles were more impacted over time and tended to be more extensive for species that typically do not dominate the vaginal tract, reflecting a more extensive need to adapt to a less familiar environment.

**Conclusions:**

This study provides insights into how vaginal and non-vaginal *Lactobacillus* species respond and adapt to a simulated vaginal environment. Overall, trends indicate high genomic stability for all species involved, with more variability in the transcriptome especially for non-dominant species of the vaginal tract.

**Supplementary Information:**

The online version contains supplementary material available at 10.1186/s12866-020-02027-8.

## Background

The increasing availability and accessibility of Next Generation Sequencing (NGS) technologies have enabled large cohort studies to aid in the establishment of population level relationships between microbes and their hosts. One such study was the Human Microbiome Project (HMP) in 2007 that allowed researchers to truly begin to understand and extensively study how microbes and microbial genes affect their human hosts, contribute to disease, and impact health [1, 2]. This project and the advent of NGS have spurred several subsequent microbiome studies, both large and small scale, into all levels of the human microbiome. The vaginal microbiome has stood out due to several of its unique properties, such as its composition, limited diversity, [2] and its role in women’s health. A hallmark study in the vaginal microbiome by Ravel, et al. showed that the vaginal microbiome of women is not only largely dominated by a single genus, lactobacilli, but often primarily consists of a single species of *Lactobacillus.* The study identified four state types (Community State Types, CSTs) that were each dominated by a select *Lactobacillus* species: *Lactobacillus crispatus, Lactobacillus iners, Lactobacillus gasseri,* and *Lactobacillus jensenii* [3]. While such a community structure stands out as unique to the vaginal microbiome, it is in alignment with long-established medical practices [4]. The results of the Ravel study were confirmed by subsequent studies, in different populations [5–7]. It should be noted that the establishment and definition of CSTs is an ongoing discussion and ethnicity does play a role [8]. However, the Ravel CSTs have been shown to be robust and are still used as a standard [9]. The combined learnings of these studies indicate that different principles shape vaginal microbiome composition and function, and that a specific approach should be considered to understand how these bacteria impact vaginal health.

A loss of lactobacilli or a dysbiosis in the vaginal microbiome typically correlates with bacterial vaginosis (BV) [10]. Treatment of BV results in a cure rate of 80% but a re-occurrence rate of 50% [11]. Additionally, BV is a contributing factor in up to 50% of visits by women to health clinics, and as such has been the focus of several studies [11]. Specifically, as the etiology of BV is unknown [12], several groups have been investigating how the microbiome fluctuates to a BV state in order to better manage this condition. However, we still have relatively little knowledge and a shallow understanding of how CSTs associate, correlate, or possibly cause disorders such as BV. Some correlations have been established and *L. crispatus* is typically considered protective against BV, while *L. iners* is considered an in-between state [13, 14]. There is limited knowledge with regards to which phylogenetic units, let alone species or strains, may provide the most promise for vaginal health and form the basis of alternative therapies. Nonetheless, drawing from the success of probiotics in the gastrointestinal tract, several groups have begun work on developing probiotic formulations for women’s health applications using *Lactobacillus* with varied results, as reviewed here [15].

In this study, we set out to examine how five distinct *Lactobacillus* species that are often identified in vaginal and intestinal environments alter their genomes and transcriptomes during long-term serial passages in a simulated vaginal fluid. We specifically selected *L. crispatus, L. gasseri,* and *L. jensenii* strains to represent CSTs associated with a healthy vaginal status [3], as well as *Lactobacillus acidophilus* and *Lactobacillus fermentum,* strains found in the intestinal cavity that have also been occasionally used commercially as potential vaginal probiotics [16, 17].

## Results

### Bacterial growth in simulated vaginal fluid

**Fig. 1:**
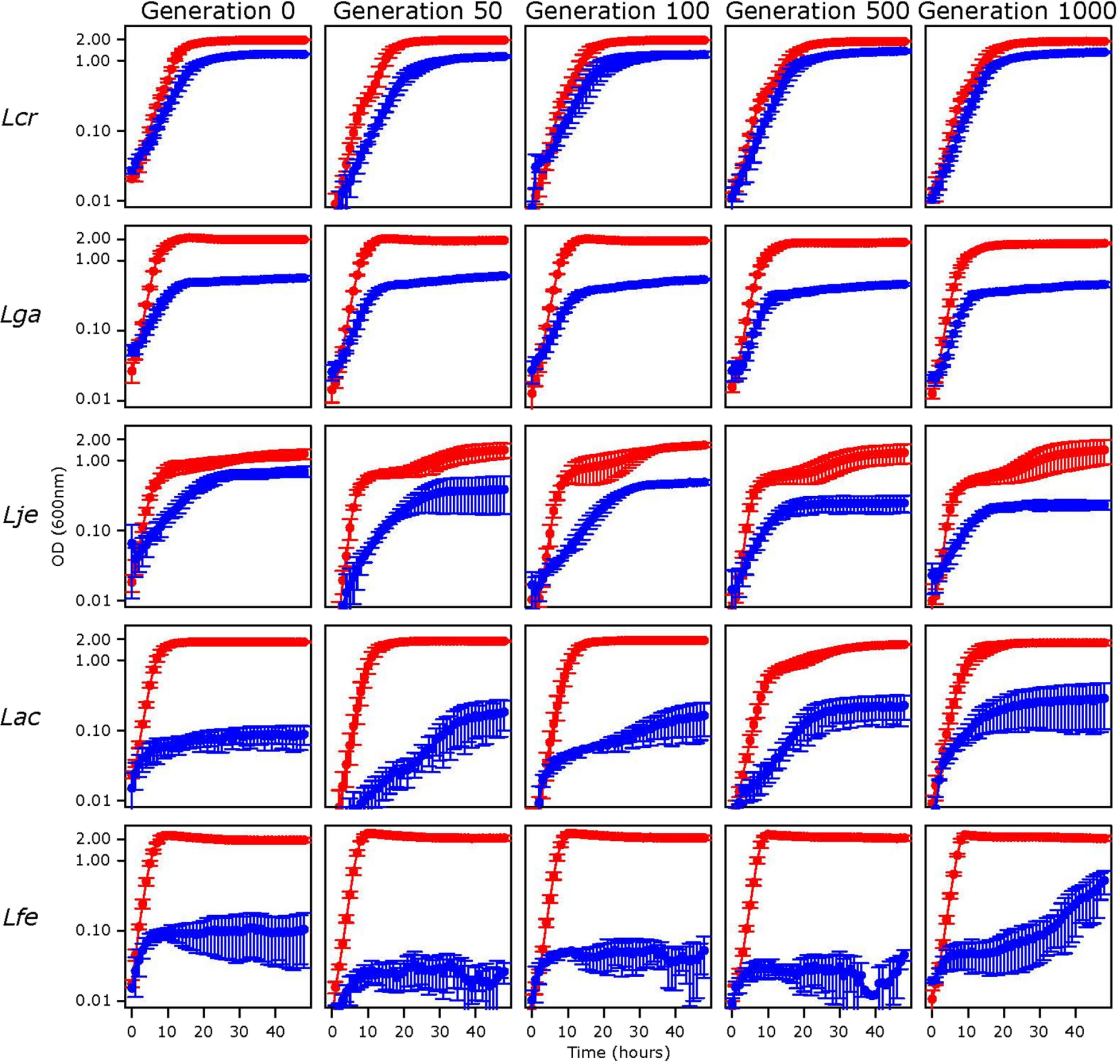
Growth in SVF and MRS. 48-h growth curves performed in SVF (blue) and MRS (red). Standard error bars are over three biological replicates. Species are organized in rows and are labelled on the left. Generations are organized by columns and are labeled at the top. Species are labelled according to Table 1

Five species were selected to study how *Lactobacillus* adapts to a simulated vaginal environment (Table 1). These species included *L. crispatus, L. gasseri,* and *L. jensenii* as representatives of healthy-associated vaginal species that frequently dominate the vaginal environment. *L. acidophilus* was included as an intestinal strain and *L. fermentum* as an outgroup. Species were passed iteratively in simulated vaginal fluid (SVF, Table 2) for 1000 generations. Growth was evaluated at generations 0, 50, 100, 500, and 1000 in both MRS (de Man, Rogosa, and Sharpe) and SVF (Fig. 1). All strains grew better in MRS than SVF. *L. fermentum* and *L. jensenii* were the best and worst growers in MRS, respectively. There were no obvious changes in growth over time in MRS for any of the strains. For SVF, *L. crispatus* grew the best, followed by *L. gasseri, L. jensenii, L. acidophilus,* and *L. fermentum. L. crispatus* and *L. gasseri* showed little change overtime in SVF. *L. acidophilus* and *L. fermentum* showed minimal growth in SVF, but there appears to be some slight improvement overtime.

**Table 1:** Strains used in this study

Species	Strain	Genome	Origin	Representation	Code	Color	Ref
*Lactobacillus crispatus*	JV-V01	ACKR00000000	Vaginal Flora	Vaginal Species	*Lcr*	Purple	[18]
*Lactobacillus gasseri*	JV-V03	NZ_ACGO	Urogenital	Vaginal Species	*Lga*	Green	[19]
*Lactobacillus jensenii*	DSM20557	NZ_AYYU01000001	Vaginal Discharge	Vaginal Species	*Lje*	Pink	[20]
*Lactobacillus acidophilus*	NCFM	NC006814	Milk	Intestinal isolate	*Lac*	Blue	[21]
*Lactobacillus fermentum*	DSM 20052	PRJNA545488	Fermented Beets	Outgroup	*Lfe*	Orange	[22]

Selection of *Lactobacillus* strains used in this study

**Table 2:** Simulated Vaginal Fluid Composition [23]

Component	Final Concentration (w/v)
Tween 80	0.1%
Ammonium citrate	0.2%
Sodium acetate	0.5%
MgSO_4_-7H_2_O	0.01%
MnSO_4_-H_2_O	0.005%
K_2_HPO_4_	0.2%
No. 3 Protease Peptone	0.3%
Urea	0.05%
Glucose	1%
Glycogen	1%
Lactic Acid	88 mM
Mucin	0.025%
Albumin	0.4%
Vitamin Solution	1X
α-Amylase	2 mU/mL

Components of vaginal media used in this study

### Genomic variations in response to iterative passages

**Fig. 2:**
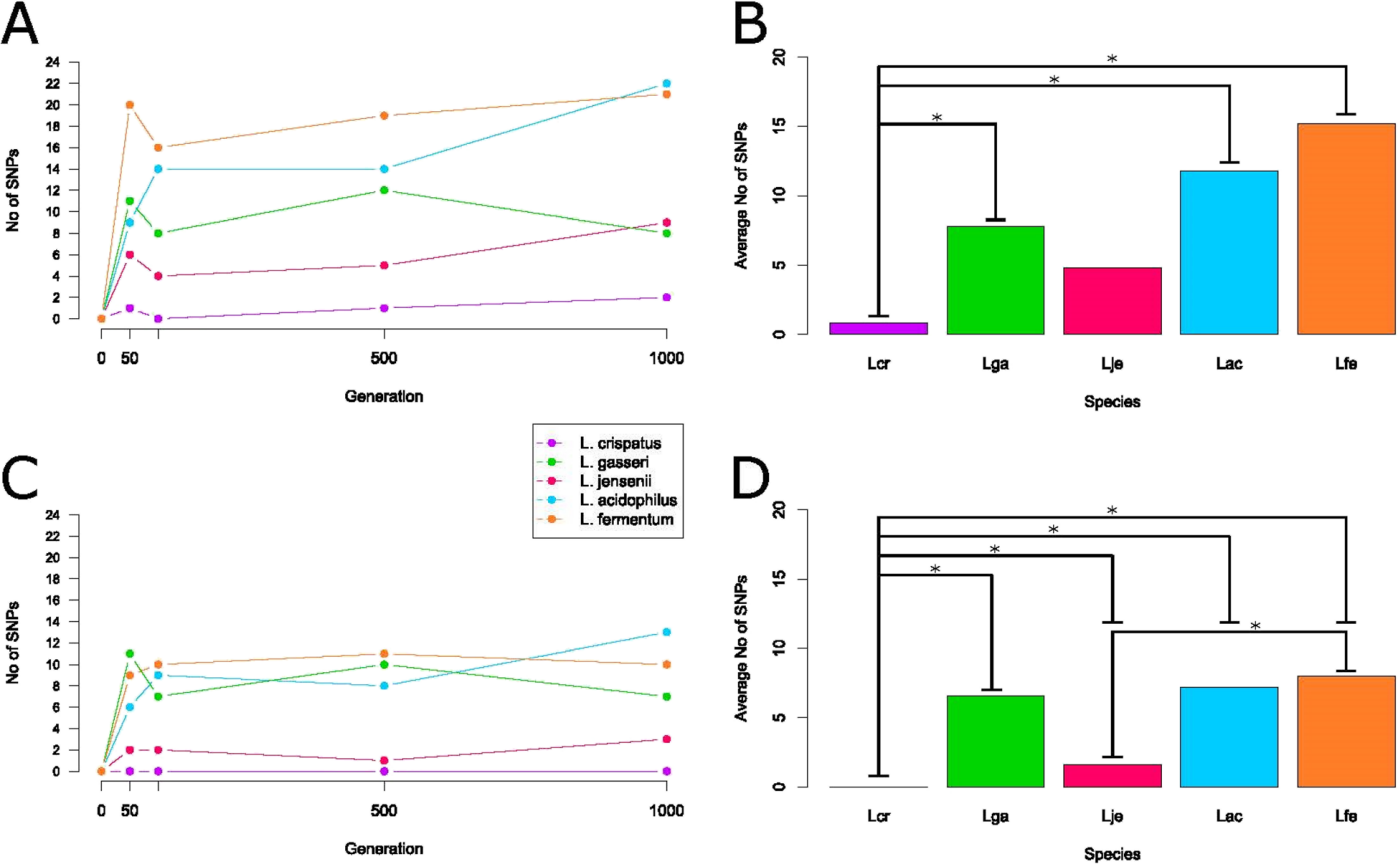
SNP Count. Total number of sites with (**a**) over 50% frequency or (**c**) 100% frequency. Number of SNPs is on the y-axis and generation is on the x-axis. Average number of SNPs with (**b**) over 50% frequency or (**d**) 100% frequency. Significance determined by a Student’s t-test (*p* < 0.05) is indicated by an asterisk (*). Species are labelled and colored according to Table 1

Next, we examined genome plasticity in SVF. We did this by mapping transcriptome reads on the reference genome to assess the occurrence of genetic mutations such as insertions, deletions, duplications and the appearance of single nucleotide variations in a population of cells using deep sequencing. SNPs were called from mRNA-Seq data using Geneious. We then filtered SNPs that were part of runs or had a coverage of less than 20 reads. Figure 2 depicts the number of SNPs over time. Both fixed SNPs (changes observed in 100% of the reads) and partial SNPs (changes observed in over 50% of reads) were mapped. *L. gasseri, L. acidophilus,* and *L. fermentum* overall had the most SNPs at 50% frequency. *L. gasseri, L. jensenii,* and *L. fermentum* had more SNPs in early generations and either remained steady or dropped in subsequent generations, contrasting *L. acidophilus* where SNPs increased over time. (Fig. 2a). *L. crispatus* had few SNPs overall. The total SNPs at 50% frequency for *L. crispatus* was significantly different compared to all other species with the exception of *L. jensenii* (Fig. 2b). *L. gasseri, L. acidophilus,* and *L. fermentum* had a high number of fixed SNPs, while *L. crispatus* and *L. jensenii* had a few or no fixed SNPs (Fig. 2c). Within the high group, *L. gasseri* had the most SNPs of the group at generation 50, *L. fermentum* at generations 100 and 500, and *L. acidophilus* at generation 1000. Of the low group, *L. crispatus* had no fixed SNPs at any time point. *L. crispatus* was significantly different from all species. *L. jensenii* was also significantly different from *L. fermentum* (Fig. 2d). Mutation rates, defined as number of SNPs per genome size per generation, can be found in Table 3.

**Table 3:** Mutation Rate

Species	# Mutations	Genome Size (bp)	Mutation Rate
*L. crispatus*	0	2,318,358	0
*L. gasseri*	7	2,011,855	2.98 × 10^− 09^
*L. jensenii*	3	1,610,429	1.86 × 10^−09^
*L. acidophilus*	13	1,993,560	6.52 × 10^− 09^
*L. fermentum*	10	1,887,974	5.30 × 10^− 09^

Mutation rate for fixed mutations at generation 1000

**Fig. 3:**
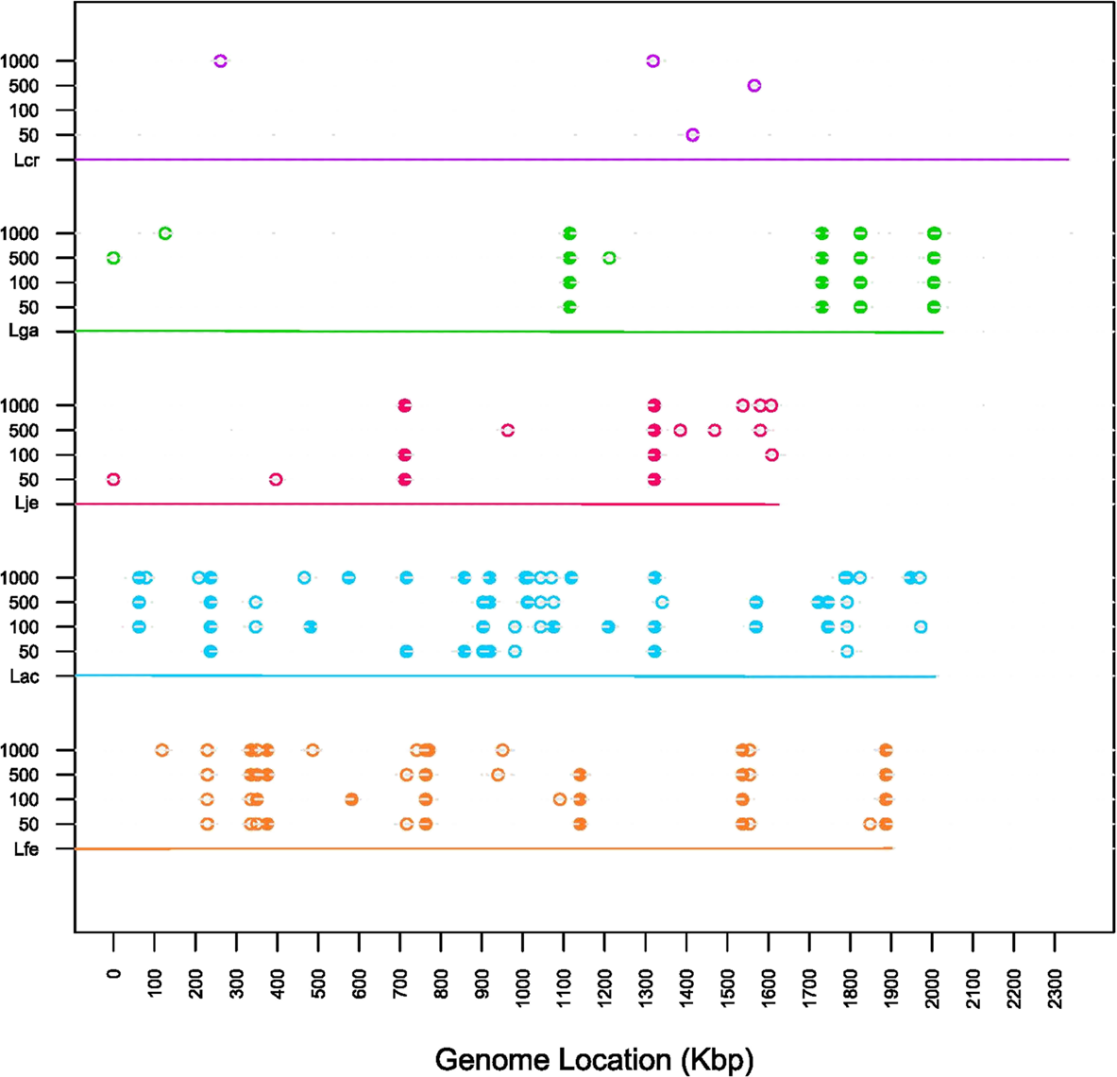
SNPs by Location. SNPs are recorded by chromosome location and generation in which they occur. Chromosome is represented by a colored line and species label is on the y-axis. Genome location is recorded on the x-axis. SNPs with over 50% frequency are represented by an open circle. SNPs with 100% frequency are represented by a closed circle. Species are labelled and colored according to Table 1

**Fig. 4:**
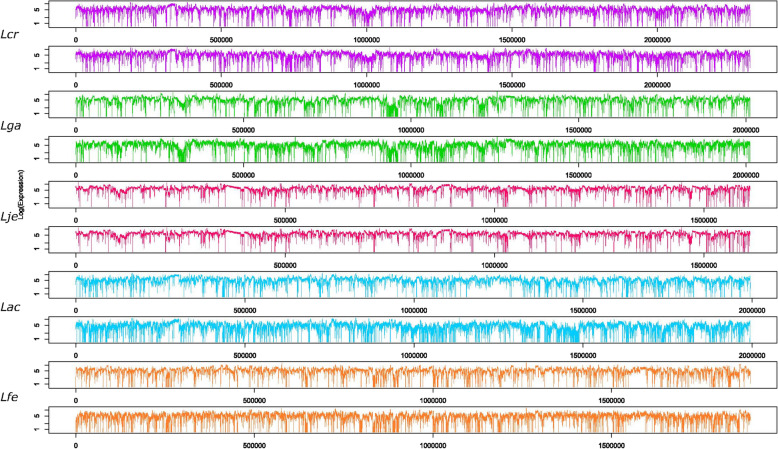
Whole Genome Transcription. mRNA coverage over the entire genome. The chromosome location (bp) is on the x-axis. The log coverage is on the y-axis. Species are labelled and colored according to Table 1. For each species, the top graph is generation 0, the bottom graph is generation 1000

After looking at SNP frequency per generation, we looked at SNPs by location and time.. Figure 3 depicts SNPs at 50% frequency (open circles) and 100% frequency (closed circles) at chromosome locations. SNPs are also separated based on the generation they were called. Once fixed, most SNPs remain in later generations. In *L. gasseri,* SNPs fixed early. For *L. acidophilus,* total SNPs and fixed SNPs increased over time. *L. fermentum* continuously gained and lost SNPs, with overall totals remaining relatively unchanged. No common hotspots were identified. We next looked to see if any large genomic events occurred, and found none in any species (Fig. 4).

### Transcriptional response to growth in simulated vaginal fluid

**Fig. 5:**
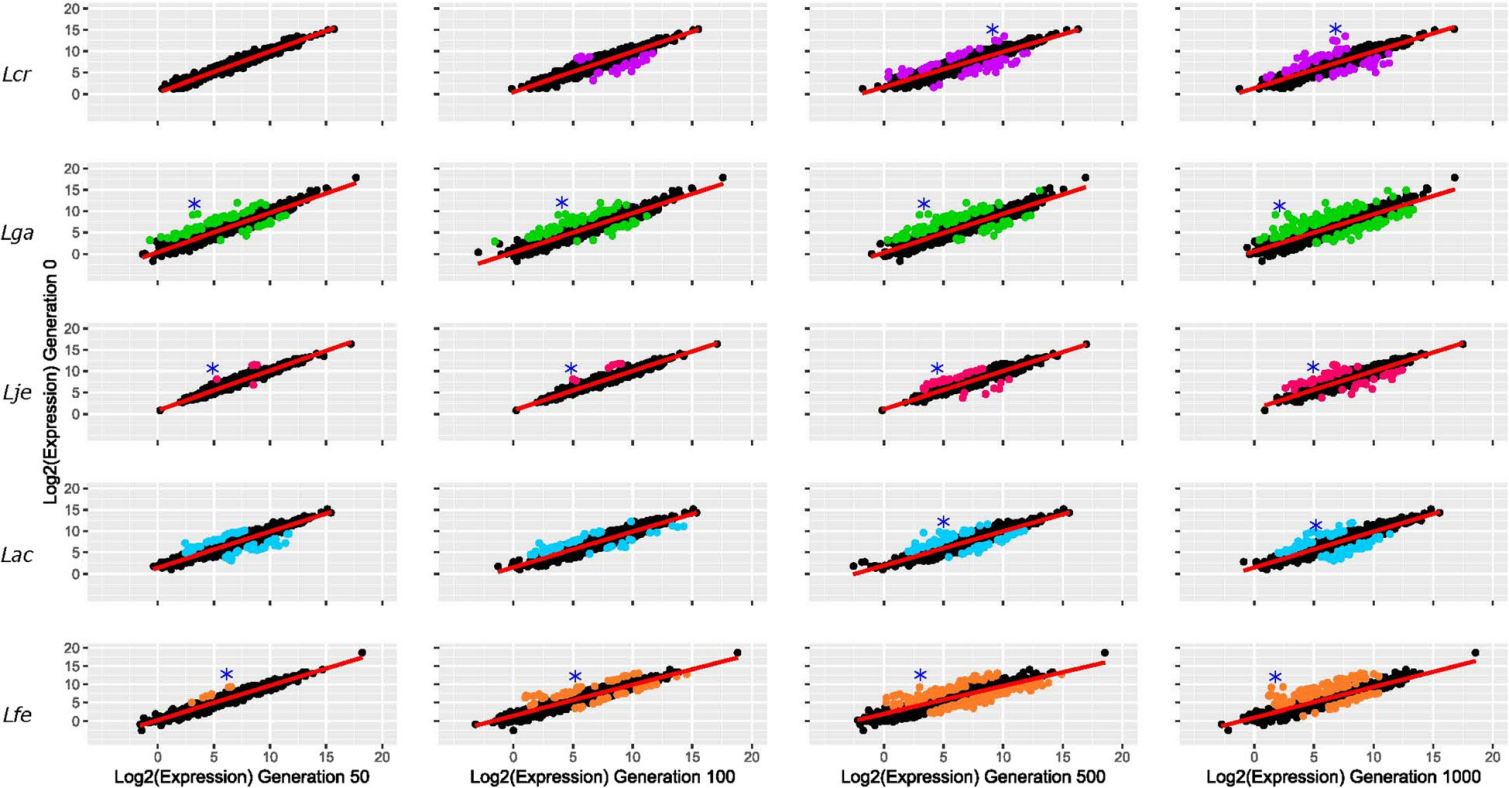
Two-way Expression. Two-way plots comparing expression between generation 0 and remaining generations. Generation 0 is along the y-axis. The x-axis denotes the compared generation. The red line is a fitted linear model. Significantly differentially expressed genes are colored. A blue asterisk (*) denotes genes that are analyzed in Fig. 7. These genes are denoted in each panel that they are significantly differentially expressed. Species are ordered by row and are labelled on the left. Species are labelled and colored according to Table 1

Following our genomic analyses, we examined how the transcriptome of each species changed after exposure to SVF. We achieved this by using generation 0 as our reference, and compared expression levels for all other generations to it (Fig. 5). Significant differences in expression are highlighted by colored dots. The greatest number of significant changes were found in *L. gasseri* and *L. fermentum*. *L. jensenii* and *L. crispatus* had the least number of changes (Table 4). In fact, there were no significant changes in expression between generation 0 and generation 50 for *L. crispatus*.

**Table 4:** Significantly Differentially Expressed Genes

Species	Generation 50	Generation 100	Generation 500	Generation 1000
***L. crispatus***	0	32	78	67
***L. gasseri***	83	98	117	191
***L. jensenii***	5	8	33	59
***L. acidophilus***	60	53	76	83
***L. fermentum***	8	67	234	137

Number of significantly differentially expressed genes compared to generation 0

**Fig. 6:**
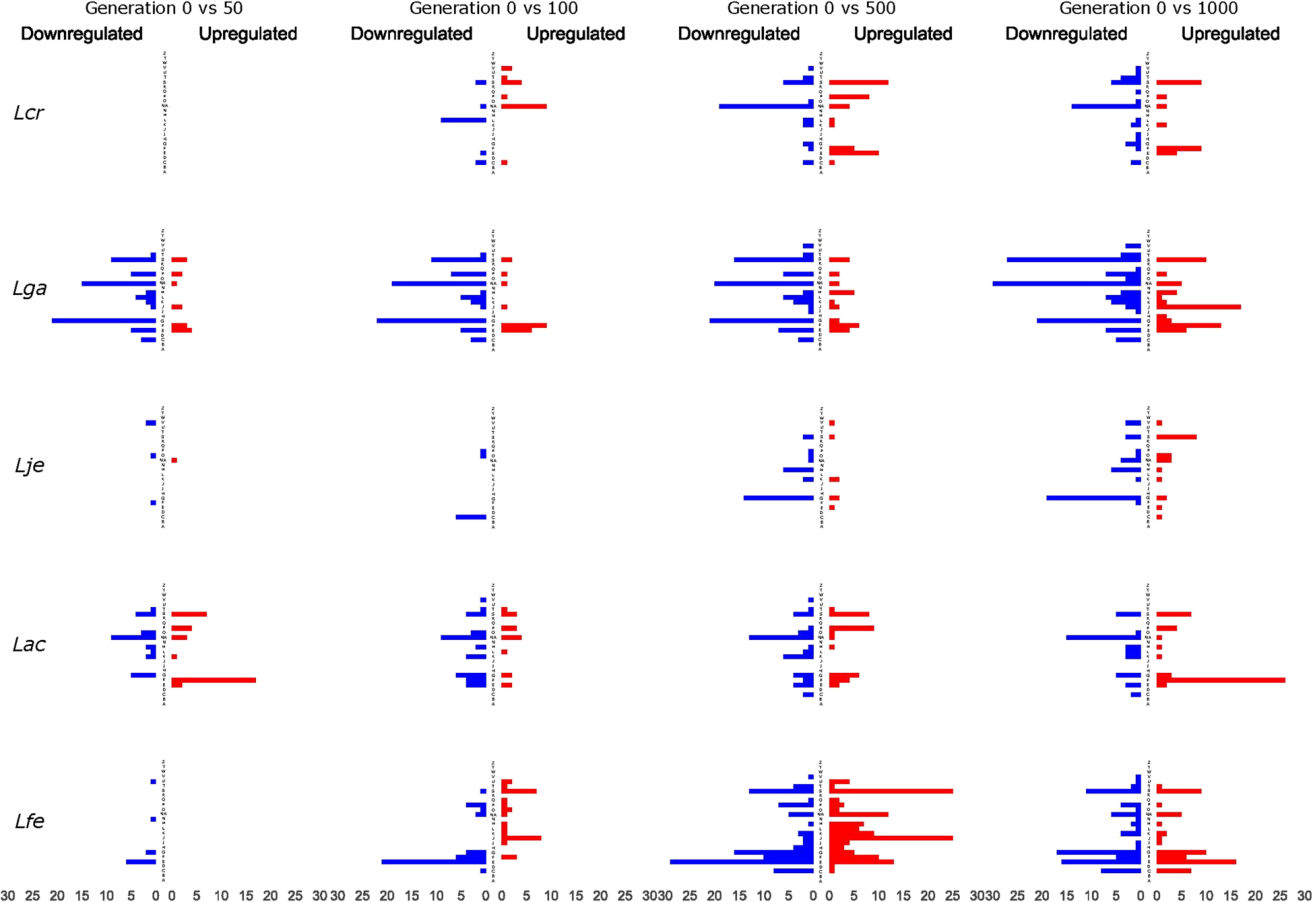
COG Regulation. Counts of upregulated and downregulated significantly differentially expressed genes by COG designation. COGs are organized alphabetically. COG order and definitions are in Additional File 1. Species are organized in rows and are labelled on the left. Generations are organized by columns and are labeled at top. Species are labelled according to Table 1

We next examined the potential function of the significantly differentially expressed genes. First, we ascribed COG designation for each significantly differentially expressed gene using eggnog. COG definitions can be found in Table S1. Then, we determined the number of genes that were upregulated or downregulated by COG designation (Fig. 6). After showing no significant changes in generation 50, *L. crispatus* began to show significant changes at tested subsequent generations, with no obvious global trend (Fig. 6). At generation 100, *L. crispatus* showed the most change in the downregulation of genes of the COG designation replication, recombination and repair (L) and the upregulation of genes with unassigned COGs (NA). Profiles for generation 500 and generation 1000 both showed downregulation of unassigned COGs (NA) and upregulation of genes with the designation of unknown function (S). Additionally, profiles for generation 500 and generation 1000 showed upregulation in amino acid transport and metabolism (E) and nucleotide transport and metabolism (F), respectively. In contrast, *L. gasseri* largely showed downregulation across its significantly differentially expressed genes (Fig. 6). Additionally, *L. gasseri* had a large number of significantly differentially expressed genes at generation 50 and gradually increased over time. Of the downregulated genes, most were of the designation carbohydrate transport and metabolism (G), function unknown (S), or unassigned a COG (NA) (Fig. 6). Of the genes upregulated in *L. gasseri,* most were a part of the nucleotide transport and metabolism (F) designation. At generation 1000, *L. gasseri* showed the most upregulation across all tested species notably for designations translation, ribosomal structure and biogenesis (J), nucleotide transport and metabolism (F), and function unknown (S). *L. jensenii* showed the least amount of significant changes and seemed most defined by downregulation over upregulation (Fig. 6). In general, most downregulation occurred at generations 500 and 1000 in carbohydrate transport and metabolism (G). Overall, *L. acidophilus* showed relatively more changes in expression than *L. jensenii,* with no obvious functional patterns (Fig. 6). The most commonly downregulated genes comprised an unassigned COG function (NA). In terms of upregulation, generation 50 and generation 1000 stand out for upregulation of nucleotide transport and metabolism (F). *L. fermentum* had the most significant changes at generations 500 and 1000 (Fig. 6). Downregulation was common in both, specifically in amino acid transport and metabolism (E) and carbohydrate transport and metabolism (G). Upregulation occurred the most in generation 500 and occurred primarily in translation, ribosomal structure in biogenesis (J), and function unknown (S).

### Transcriptional differences between species

**Fig. 7:**
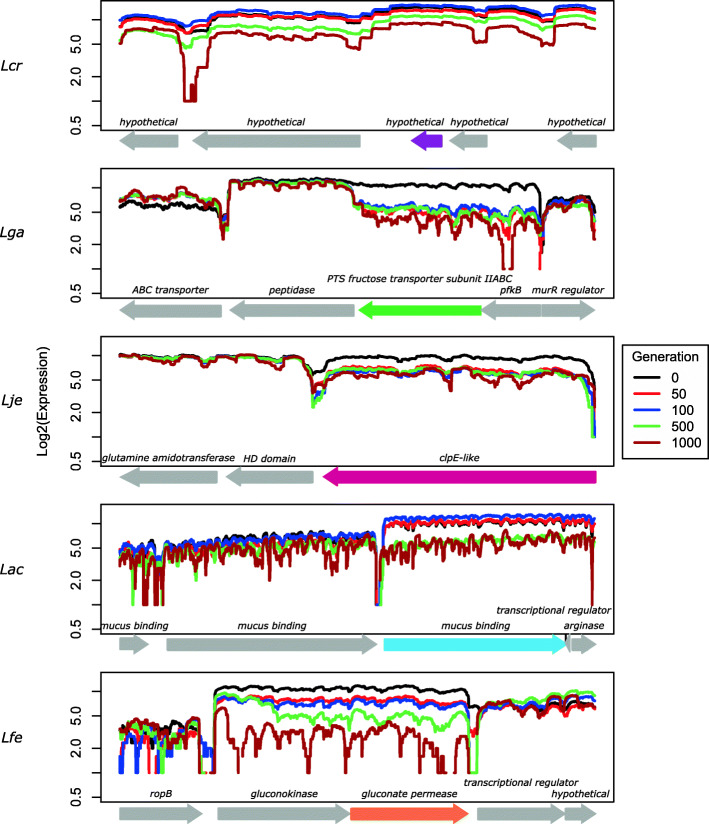
Locus Transcription. Expression of select locus for each species. Locus of interest is colored according to Table 1. Two loci upstream and downstream of the locus are shown and colored in gray (*L. jensenii* was at the end of a contig, so only loci upstream are shown). Expression is on the y-axis; position is on the x-axis. Arrows denote gene direction. Species are organized in rows and are labelled on the left. Species are labelled according to Table 1

Finally, we highlight one uniquely differentially transcribed locus from each species (Fig. 7). These genes were selected from Fig. 5 based on their consistent significance, and have been marked by an asterisk (*). *L. crispatus* and *L. fermentum* loci both had decreasing transcription as generations passed. *L. gasseri* and *L. jensenii* had genes that downregulated early and remained around the same level. For *L. crispatus*, a hypothetical gene was selected. This gene was one of three hypothetical genes that were significantly downregulated at generations 500 and 1000, as compared to generation 0. From *L. gasseri*, a PTS fructose transporter subunit II ABC locus is depicted (Fig. 7). It is one of two genes that were consistently downregulated in all generations compared to generation 0 and was easily distinct in two-way plots (Fig. 5) (the other *pfkb*). A clpE-like protein locus is depicted for *L. jensenii* (Fig. 7). It was one of the few genes that showed significant expression between generations 0 and 50, and remained significantly differentially expressed in subsequent generations (Fig. 5). Its expression profile for generations 50, 100, 500, and 1000 was quite consistent. *L. fermentum* had many genes significantly differentially expressed across generations and a few were expressed in subsequent generations (Fig. 5). One such gene was *gluconate permease*, which was downregulated in all generations compared to 0; its expression profile is shown in Fig. 7. *L. acidophilus* also had many genes that were significantly differentially expressed each generation. A mucus binding protein locus is shown in Fig. 7. It was significantly downregulated in generation 500 and generation 1000 but was not significantly differentially expressed in generation 50 and generation 100 (Fig. 5).

## Discussion

In this study, we sought to understand how *Lactobacillus* species adapted to a simulated vaginal environment over time. Experimental evolution and adaptation have been studied in bacteria for several species under various conditions through iterative passages [24–27]. Here, we examined how vaginal and non-vaginal strains from five distinct *Lactobacillus* species adapted to iterative passages for 1000 generations in SVF. Strain selection was guided by several recent studies highlighting their occurrence and potential role in women’s health. From the establishment of CSTs, *L. crispatus, L. gasseri,* and *L. jensenii* strains were included as representatives of normal microbiomes. We strategically elected to forego *L. iners* due to its controversial status, potentially linked to its different properties of the other CST species, as well the practical need this species has to grow in blood, rendering it an impossible comparative growth outlier [14, 28]. We chose to include *L. acidophilus* as a representative of an intestinal strain and potential vaginal contaminant to compare and contrast vaginal vs. intestinal isolates. Additionally, *L. acidophilus* is a well-known gut probiotic and has been proposed and even commercialized as a vaginal probiotic [29, 30]. Finally, *L. fermentum* was used as an outgroup --as a food isolate which can also be found in the human gastrointestinal tract [GIT] -- though this species is also a candidate for women’s health applications [31, 32]. Care was taken to ensure that the strains selected were isolated from the above environments, were available through strain collections, had reference genomes readily available, and had been documented in the literature, including some model strains widely used to represent the species of interest. However, it is important to note that there is broad diversity in *Lactobacillus* in general and select *Lactobacillus* species in particular, with some species showcasing high genomic homogeneity (e.g. *Lactobacillus acidophilus*) and others displaying broad genetic diversity (e.g. *Lactobacillus crispatus*). This means that various strains and isolates within species of interest could behave differently, so future studies should investigate the impact of strain diversity on environmental fitness and identify the genetic determinants of niche adaptation and markers of health.

To date, the primary concept underlying protection of the vaginal environment by *Lactobacillus* species colonization is competitive exclusion [33], which presumably hinges on the ability of these strains to survive and grow in the vaginal environment. Therefore, we began our analyses by comparing strain growth in MRS (primary defined medium for *Lactobacillus* species growth in a laboratory setting) and SVF [23]. As anticipated, all strains grew well in MRS and did not showcase altered growth in this medium over the time course of the experiment. As expected, strains grew better in the *Lactobacillus*-optimized medium, MRS, than they did in SVF (Fig. 1). *L. fermentum* grew the best, while *L. jensenii* grew the least in MRS. In terms of SVF, the vaginal representative strains grew better than their intestinal counterparts. Over time, *L. crispatus* and *L. gasseri* showed relatively little growth change across generations, presumably because they are already adapted to the environmental conditions provided by this simulated vaginal fluid. Towards the latter stages of the experiment, it appears the growth of *L. acidophilus* increases, though the improvement may be relatively limited and growth benefits would likely warrant a more extensive period of time.

Having carried out iterative passages over time and sampled the strains at select time points through the 1000 generations of growth, we next determined their genome sequence to monitor genetic plasticity over time. Based on similar studies with *Escherichia coli,* and the fact that we performed experiments with broth cultures, we anticipated adaptation at an early passage stage, with less modifications at the latter stages [34], and our results suggest that indeed the bulk of fixed mutations did occur by generation 50, though some SNPs appeared thereafter (Fig. 2). At 50% frequency, *L. gasseri, L. acidophilus,* and *L. fermentum* overall had the most SNPs (Fig. 2a). The *L. acidophilus* SNPs increased over time, in a pattern somewhat consistent with the aforementioned increase in growth in SVF over the course of the experiment. Intriguingly, *L. crispatus* had very little SNPs at 50% frequency (Fig. 2a), and was significantly different from all but *L. jensenii* (Fig. 2b). For fixed SNPs, the five species distributed in the same two groups, with *L. gasseri, L. acidophilus,* and *L. fermentum* encompassing the high-SNP group, and *L. crispatus* and *L. jensenii* constituting the low group (Fig. 2c). *L. crispatus* had no fixed SNPs, reflecting the low number of SNPs at 50% frequency and was once again significantly different from all species (Fig. 2d). From here, we calculated the mutation rates found in Table 3. With the exception of *L. crispatus*, mutations rates were similar to those reported for *Lactobacillus rhamnosus*, but slightly higher than those in *E. coli* [35, 36]. None of the strains took on a mutator phenotype [25, 35, 37].

After enumerating the number of SNPs per generation, we next examined how individual SNPs change over time and by location (Fig. 3). Due to the level of magnification shown on the figure, some SNPs are indistinguishable from others, as in *L. gasseri.* One gene, *rpn family recombination-promoting nuclease/putative transposase,* had four SNPs occur in subsequent generations, all of which resulted in synonymous changes. Here, it can be seen in most strains that once a SNP is fixed, it typically remains in successive generations. This is most noticeable in *L. gasseri,* whose majority of SNPs fix early and remain fixed. In contrast, *L. fermentum* continuously acquires and loses SNPs, although overall numbers remain fairly steady. This diversity reflects the presence of a mixed microbial population and the rise and fall of various genotypes with no apparent gain of fitness over the course of the experiment. This contrasts with *L. acidophilus,* which increases in total SNPs and especially fixed SNPs in later generations, which is noteworthy and could reflect a functional benefit acquired over time by some members of the mixed population. Overall, mutations occurred throughout the chromosomes, at various locations across species with no common hot-spot, similar to previous studies [35]. It is noteworthy that we did not detect any large insertion, deletion, duplication or genomic re-arrangement in any of these five species, perhaps reflecting the overall genetic stability of these strains in these conditions (Fig. 4). In fact, evolution studies in other lactic acid bacteria species (*L. rhamnosus* and *Lactococcus lactis*) identified large deletions, which was attributed to insertion sequence (IS) elements [35, 37, 38].

After determining the genomic changes over time, we next examined how the transcriptome changed over time to assess whether the regulation of select transcripts reflected adaptation to SVF composition and substrates. Iterative passages and expression experiments were performed in SVF only. Due to the extent of differences between MRS and SVF, we anticipate that the changes described in this study result from the change in the growth conditions we tested. We began by comparing expression levels between generation 0 and subsequent generations for each species (Fig. 5). Overall, relatively few transcripts showed extensive differential expression. However, there were significant differences in expression for each species (Fig. 5). Notably, there were no significant changes in expression between generation 0 and generation 50 for *L. crispatus* (Table 4).

After determining which genes were significantly differentially expressed, we investigated the potential functions of these genes. Each species had unique changes in their transcriptome, but patterns did emerge. Overall, downregulation was common, which perhaps reflects the rich composition of MRS, in which more substrates are available. Across species, the COG designation (G) carbohydrate transport and metabolism was often downregulated and (F) nucleotide transport and metabolism was upregulated. Additionally, changes occurred often in the unknown function (S) and unassigned (NA) groups. These changes have been noted in other studies as well [23]. In contrast to genetic changes, which primarily occurred early on (most SNPs detected by generation 50), it appears most transcriptional impact was detected at later generations, suggesting a more significant impact over time on the transcriptome than the genome (Table 4).

Figure 7 depicts a select locus highly impacted for each species and the corresponding transcriptional profile at each analyzed generation. Each gene represented a different COG: a hypothetical gene that returned no known domains from *L. crispatus* (unassigned COG), a *PTS fructose transporter subunit II* from *L. gasseri* (COG group (G))a *clpE-like* gene from *L. jensenii* (COG group (O)),and a *gluconate permease* from *L. fermentum* (COG group (E) and (G)). The locus from *L. acidophilus* was perhaps the most interesting. This was the only highlighted locus that also had an acquired nonsynonymous SNP (Fig. 7)*,* reflecting a convergence between the transcriptional response and genetic mutation pattern over the course of the experiment. This was the only gene to show subsequent SNPs in the same location and a correlated significant change in expression. The SNP was a change from an A to a C (transversion) and caused an amino acid change from a glutamic acid to alanine. Intriguingly, this SNP was acquired in generation 500 and remained in generation 1000. In correlation, there was no significant change of expression until generation 500, where the expression changed from not significantly expressed to significantly downregulated. The locus (LBA1020) is annotated as a mucus binding protein. It had no homologs (threshold of 40% similarity) in any of the other *Lactobacillus* strains in this study. Additionally, no other works reference this locus. Despite this, it is highly interesting because mucin is added to SVF, suggesting direct adaptation.

We hope this study will serve as a foundation for follow-up studies that will investigate a more diverse set of strains and identify key genetic drivers of vaginal vs. intestinal niche colonization. We have shown that vaginal lactobacilli are well adapted and that non-vaginal lactobacilli may yet still adapt to a vaginal environment, as exemplified by *L. crispatus* and *L. acidophilus,* respectively. Overall, we did not identify large changes in phenotype over the experimental time course, nor did we identify consistent markers among vaginal lactobacilli. While this may seem unexpected, it is consistent with previous studies. Indeed, it has been established that *Lactobacillus* species adapt to their niche through several genomic changes, such as genome decay [39, 40]. Often times, these genomic changes reflect their environment and are shared by other lactobacilli in the same niche. Interestingly, signature genomic features have not been identified among vaginal lactobacilli, indicating that vaginal adaptation occurred multiple times independently in a lineage-specific manner [40]. A recent study went a step further and evaluated not only the difference in species niche adaptation, but also strain adaptation. This study compared vaginal and intestinal strains of *L. crispatus* and *L. gasseri* and noted significant changes when exposed to the opposite environment [23]. This implies that vaginal adaptation occurs at the strain level within these species. It is also indicative of why *L. crispatus* is part of a CST and performed well in SVF, whereas *L. acidophilus* is not a CST and did not perform well. Additionally, these evolutionary trends highlight the importance of understanding how different phylogenetic units interact and adapt to a niche when considering probiotic candidates. In our study, the most notable changes were identified in the transcriptome. Other studies have also delved into the vaginal transcriptome. One study evaluated various transcriptional response to BV in order to clarify health impact of vaginal lactobacilli [41]. The study noted several escape mechanisms of BV from antibiotics, with an increase in CRISPR-Cas activity in the vaginal species [41]. This supports the hypothesis that phages may be playing a larger role than originally thought in women’s health [42]. Further characterizing how vaginal species adapt to the vaginal environment and provide host protection will be imperative to establish alternative therapies, such as probiotics, to BV, especially in cases that do not resolve with antibiotics. We also anticipate that our understanding of the vaginal microbial community will continue to expand over time, and will predictably reveal more diversity between individuals, within individuals, and likely unravel more strain diversity within species of interest, as well as unique cases where non-canonical bacterial patterns are observed (e.g. cases where *L. acidophilus* or *L. fermentum* may be un-expectedly enriched). Overall, our results further highlight the need to further advance our understanding of the vaginal microbiome, and of the *Lactobacillus* determinants that impact women’s health.

## Conclusions

In conclusion, we established how vaginal and non-vaginal strains genomically adapt to a simulated vaginal environment. Previous directed evolution experiments in LAB showed niche-specific and stress adaptations after 1000 generations, with defined phenotypes [35, 37], however we did not see strong changes in phenotype. The only correlation between passage, genomic change, and transcription change was the mucin binding locus in *L. acidophilus*. As this locus has not been fully characterized, it is a potential feature of interest for future studies. Additionally, we did not see the previously reported large chromosome changes or mutator phenotypes [35, 37]. We did see greater growth in SVF and less mutations in our vaginal strains, specifically *L. crispatus*, as compared to the non-vaginal strains.

Overall, vaginal strains showed better growth in SVF, fewer genetic alterations and modest transcriptional changes compared to non-vaginal strains. Additionally, while performing relatively poorly at initial exposure to SVF, non-vaginal strains did not greatly improve over time in a simulated vaginal environment. They had modest growth gains, relatively few genetic changes primarily consisting of SNPs, and limited transcriptional responses, reflecting media composition differences. This implies that the differences between vaginal strains and non-vaginal strains is not non-significant. These findings open new avenues to investigate and characterize members of the vaginal microbiome and inform the genetic and functional assessment of strains of potential value for enhancing women’s health, with preference for vaginal over intestinal isolates for candidate probiotics.

## Methods

### Strain selection

In order to evaluate the adaptive response to the vaginal environment, five strains from five distinct species of *Lactobacillus* were selected: *L. crispatus* JV-V01, *L. gasseri* JV-V03, *L. jensenii* DSM-20557, *L. acidophilus* NCFM, and *L. fermentum* DSM 20052. These strains represent vaginal species (*L. crispatus, L. gasseri,* and *L. jensenii*), intestinal contaminants (*L. acidophilus*), and a phylogenetic outgroup (*L. fermentum*) [43]. Table 1 contains information on each strain, encompassing their source of isolation as well as genomic information. *L. acidophilus* and *L. gasseri* both had publically available genomes [21]. The *L. fermentum*’s genome was completed as previously described [22]. The *L. jensenii* genome, was sequenced at CoreBiome (St. Paul, MN). Briefly, samples were isolated using MO Bio PowerFecal (Qiagen) for high-throughput on QiaCube (beading beating 0.1 mm). DNA quantification was determined using Invitrogen’s Qiant-iT Picogreen dsDNA Assay. Library preparation was performed using the Nextera Library Prep kit (Illumina). Sequencing was carried out using Illumina NextSeq 500/550 High Output V2 kit using paired-end reads (2 × 150 reads). Reads were then filtered (Q-score < 20; length < 50) and adapter sequences were removed using cutadapt (v1.15). Sequences were then assembled using SPAdes (v3.11.0) and QUAST (v4.5). We then annotated the *L. jensenii* genome using RAST. The *L. crispatus’s* genome was sent to the Roy J. Carver Biotechnology Centre from the University of Illinois (Urbana-Champaign, IL) for DNA extraction and sequencing. Briefly, library preparation for short reads was completed using the Hyper Library construction kit from Kapa Biosystems (Roche). It was then quantified using qPCR before sequencing on a MiSeq flow cell using a MiSeq 500-cycle sequencing kit (v2). Demultiplexing was achieved using bcl2fastq (v2.20) Conversion Software (Illumina). Long reads were generated by converting 600 ng of DNA to barcoded Nanopore libraries suing the Rapid Barcoding kit SQK-RAD004. Sequencing then occurred using a GridION × 5 sequencer on SpotON R9.4.1 FLO-MIN106 flow cells. Guppy (v1.8.1) was used for base-calling and Porechops (v0.2.3) was used for demultiplexing and adapter removal. Reads were then assembled using the Unicycler assembler (v0.4.4) and annotated using Prokka (v1.12).

### Growth conditions

Our lab recently developed a simulated vaginal fluid to mimic the vaginal environment and conditions in a laboratory setting (SVF, Table 2) [23]. Based off of a semi-defined medium, SVF incorporates nutrients and enzymes common to the vaginal environment, such as glycogen, α-amylase, and a pH of 4.5 [23]. Directed evolution was achieved by passaging these strains for 1000 generations in preconditioned (24 h in anaerobic conditions at 37 °C) SVF. We assessed adaptation at five generations: 0, 50, 100, 500, and 1000. These generations were used throughout this study. Generation 0 is defined as the first passage from freezer stock, sampled from a broth culture, in MRS (de Man, Rogosa, and Sharpe, Fisher Scientific DF0881-17-5 ) to growth in SVF and represents the first naïve exposure to a vaginal environment. This serves as our reference point for all other time points analyzed.

Growth medium was preconditioned anaerobically at 37 °C for 24 h before inoculation. At the time of the daily inoculation, α-amylase (2 mU/mL) was added. Passages were inoculated 1% (vol/vol) from the previous day. Strains were grown for 24 h anaerobically at 37 °C, and passed iteratively for a total of 1000 generations (6.6 generations/transfer for 151 transfers). After reaching a generation of interest, cultures were stocked during the following inoculation.

For growth curves, MRS and SVF were preconditioned for 24 h with cysteine (0.05%, wt/vol). Cultures were inoculated from freezer stocks (of the desired generation) into preconditioned SVF (MRS for generation 0) and grown for 24 h anaerobically at 37 °C. 96-well microplates were inoculated 1% (v/v) from the overnight culture in triplicate. Each well contained 200 μL of SVF or MRS. A clear Thermalseal film was used to then seal the plates. Using a Floustar Optima microplate reader (BMG Labtech, Cary, NC), plates were read at 37 °C, 600 nm for 48 h. Growth curves were completed three times. Analyses were carried out between the biological replicates (averages of the technical replicates).

### Transcriptome analysis

Cells were inoculated from freezer stock (of the desired generation) and grown overnight in preconditioned SVF (MRS for generation 0) anaerobically at 37 °C. Cells were then passed (1% v/v) and grown to mid-log phase and flash frozen. Zymo Direct-Zol Miniprep kit (Zymo Research, Irvine, CA) was used to extract total RNA, as previously described [44]. mRNA library preparation and sequencing were completed at the Roy J. Carver Biotechnology Centre from the University of Illinois (Urbana-Champaign, IL) using an Illumina HiSeq2500. Data was uploaded into Geneious (v. 11.1.5, https://www.geneious.com). Read processing included trimming (error probability limit of 0.001) and filtering (length extracted 28–150 nt). Reads were then mapped to the reference genome using Bowtie2 [45].

### Data analysis

Statistical analyses were completed using R (v3.5.1). Expression levels were calculated in Geneious (count as partial matches/CDS). Levels were compared using Geneious’s built in method (transcripts/median of gene expression rations). Gene significance was determined using the Geneious metrics Differential Expression Absolute Confidence (>|6|) and the Differential Expression Log2 Ratio (>|2|). Significantly differentially expressed genes were hand curated from Geneious and then assigned Clusters of Orthologous Groups (COGs) by eggnog-mapper, based on eggnog 4.5 data [46, 47]. SNP variations were calculated using the Geneious tool Find Variations/SNPs with default settings. Generation 0 was set as the base reference for each species for SNP calling. SNPs were additionally filtered by removing SNPs generated through runs and a coverage cutoff of 20. Chromosomal location of SNPs from non-closed genomes was determined by ordering contigs by size. Mutation rates were determined by total number of SNPs per genome size per generation. Student’s t-test (α = 0.05) was used to determine significance between groups.

## Supplementary Information

**Additional file 1 Table S1**. COG Description. COG symbols and their descriptions.

**Additional file 2 *Lactobacillus crispatus*** **differential expression.**

**Additional file 3 *Lactobacillus gasseri*** **differential expression.**

**Additional file 4 *Lactobacillus jensenii*** **differential expression**.

**Additional file 5 *Lactobacillus acidophilus*** **differential expression.**

**Additional file 6 *Lactobacillus fermentum*** **differential expression.**

**Additional file 7 *Depth of sequencing coverage across species and timepoints.***

## Data Availability

The datasets generated in this study are available at NCBI under BioProject PRJNA600659. Data is available at https://www.ncbi.nlm.nih.gov/sra/PRJNA600659.

## Abbreviations

NGS: Next Generation Sequencing
HMP: Human Microbiome Project
CSTs: Community State Types
BV: Bacterial Vaginosis
SVF: Simulated Vaginal Fluid
MRS: de Man, Rogosa, and Sharpe
IS: Insertion Sequences
