# Supplementary material for: Adaptive response to iterative passages of five Lactobacillus species in simulated vaginal fluid
Source: BMC Microbiol. 2020 Nov 10;20:339. doi: 10.1186/s12866-020-02027-8 (PMC7653816; doi:10.1186/s12866-020-02027-8)
Supplement: Supplementary file 1 — Additional file 1 Table S1. COG Description. COG symbols and their descriptions. [file 12866_2020_2027_MOESM1_ESM.pdf]

Additional File 1| COG Description.

COG Symbol	Description
NA	Unassigned
Z	Cytoskeleton
Y	Nuclear structure
W	Extracellular structures
V	Defense mechanisms
U	Intracellular trafficking, secretion, and vesicular transport
T	Signal transduction mechanisms
S	Function unknown
R	General function prediction only
Q	Secondary metabolites biosynthesis, transport, and catabolism
P	Inorganic ion transport and metabolism
O	Post-translational modification, protein turnover, and chaperones
N	Cell motility
M	Cell wall/membrane/envelope biogenesis
L	Replication, recombination and repair
K	Transcription
J	Translation, ribosomal structure, and biogenesis
I	Lipid transport and metabolism
H	Coenzyme transport and metabolism
G	Carbohydrate transport and metabolism
F	Nucleotide transport and metabolism
E	Amino acid transport and metabolism
D	Cell cycle control, cell division, chromosome partitioning
D	Energy production and conversion
B	Chromatin structure and dynamics
A	RNA processing and modification

COG symbols and their descriptions.
